# Th1 cytokines in conjunction with pharmacological Akt inhibition potentiate apoptosis of breast cancer cells *in vitro* and suppress tumor growth *in vivo*

**DOI:** 10.18632/oncotarget.27556

**Published:** 2020-07-28

**Authors:** Loral Showalter, Brian J. Czerniecki, Gary K. Koski

**Affiliations:** ^1^Department of Biological Sciences, University Esplanade, Kent State University, Kent, Ohio, USA; ^2^Department of Breast Oncology, H. Lee Moffitt Cancer Center and Research Institute, Tampa, FL, USA

**Keywords:** breast cancer, Akt kinase, immunotherapy

## Abstract

Targeted drug approaches have been a major focus for developing new anticancer therapies. Although many such agents approved in the last 20 years have improved outcomes, almost all have underperformed expectations. The full potential of such agents may yet be obtained through novel combinations. Previously, we showed that anti-estrogen drugs combined with a dendritic cell-based anti-HER-2 vaccine known to induce strong Th1-polarized immunity dramatically improved clinical response rates in patients with HER-2^pos^/ER^pos^ early breast cancer. Here, we show that the small molecule Akt antagonist MK-2206, when combined with the Th1 cytokines IFN-gamma and TNF-alpha, maximize indicators of apoptotic cell death in a panel of phenotypically-diverse human breast cancer lines. These findings were mirrored by other, structurally-unrelated Akt-targeting drugs that work through different mechanisms. Interestingly, we found that MK-2206, as well as the other Akt antagonist drugs, also had a tendency to suppress Th1 cytokine expression in stimulated human and murine lymphocytes, potentially complicating their use in conjunction with active immunotherapy. After verifying that MK-2206 plus IFN-gamma could show similar combined effects against breast cancer lines, even in the absence of TNF-alpha, we tested in a rodent HER-2^pos^ breast cancer model either a HER-2-based DC vaccine, or recombinant IFN-gamma with or without MK-2206 administration. We found that for MK-2206, co-administration of recombinant IFN-gamma outperformed co-administration of DC vaccination for slowing tumor growth kinetics. These findings suggest a combined therapy approach for Akt-targeting drugs that incorporates recombinant Interferon-gamma and is potentially translatable to humans.

## INTRODUCTION

Once existing only at the margins of cancer treatment, immunotherapy has now gained a strong claim as a distinct and accepted treatment modality, taking its place among the established approaches of surgery, radiation, cytotoxic agents and targeted drugs [1]. Our own group has pioneered the use of IL-12-secreting, Th1-polarizing dendritic cells (DC) pulsed with HER-2 peptides for the treatment of early breast cancer (ductal carcinoma *in situ*; DCIS) in the neoadjuvant setting [2–4]. In the initial trial, about 18% of vaccinated subjects had no remaining disease at the time of surgery, which we define as a pathological complete response (pCr) [2]. About half of the remaining subjects also demonstrated strongly diminished HER-2 expression in post-vaccine surgical specimens, as compared with the pre-vaccine diagnostic biopsy. Interestingly, post-vaccine recruitment of CD4^pos^ T cells into peritumoral areas strongly predominate over CD8^pos^ T cells, suggesting an effector mechanism dependent at least in part on Th cells. Consistent with this notion, we and others have found that paired Th1 cytokines IFN-γ and TNF-α induced senescence and/or apoptosis *in vitro* for a variety of cancer cell lines (both human and murine) [5–7]. We also showed that in many cases these cytokines could drive down the expression of HER family members on the surface of breast cancer cells [6]. Thus Th1 cytokines mimic *in vitro* many of the effects of vaccination. When examining patient characteristics that predisposed toward pCR in response to DC vaccination, we observed that subjects with ER^pos^ DCIS had only a 5% pCR rate, while their ER^neg^ counterparts had a 30% pCR rate [3]. Subsequent *in vitro* studies showed that ER^pos^ BT-474 cells were relatively resistant to Th1 cytokines while ER^neg^ SK-BR3 cells were more sensitive [8]. However, addition of anti-estrogen drugs to cytokines for BT-474 cells had about the same impact as cytokines alone on SKBR3, i. e. the drugs that blocked estrogen signaling appeared to sensitize estrogen-dependent cells to the Th1 cytokines. This observation prompted a new clinical trial where a brief course of anti-estrogen therapy was supplied to ER^pos^ DCIS subjects concurrent with vaccination. In this second trial, pCRs of ER^pos^ subjects increased from 5% to about 30% such that their rates were now no longer statistically different from their ER^neg^ counterparts [9]. This study showed that combining vaccination with small molecule drugs capable of inhibiting signaling pathways associated with maintenance of an oncogenic phenotype could dramatically enhance clinical response rates. It also suggested that *in vitro* testing of such small-molecule targeted drugs for enhanced anti-tumor activity, when combined with Th1 cytokines, could function as an effective screen for identifying combinations with the potential to demonstrate activity *in vivo*.

The search for small molecule inhibitors that selectively target proteins critical for the maintenance of an oncogenic phenotype has led to the development of a number of drugs that have been tested in clinical trials. In the past 15 years, drugs such as sunitinib, lapatinib, palbociclib, and a host of others have gained approval and now form part of our armamentarium for dealing with a variety of malignancies [10–12]. Our success in using estrogen antagonist drugs to improve vaccine efficacy has led us to look for additional small molecule inhibitors that may work in conjunction with immunotherapy, particularly those reliant on strong Th1 immunity, with the goal of improving patient outcomes. It is important to identify additional compounds because whereas anti-estrogen drugs greatly enhanced vaccine efficacy in ER^pos^ subjects, the overall pCR rate for ER^pos^ and ER^neg^ is still only 30%. Pharmacological suppression of additional oncodrivers in conjunction with immunotherapy is likely to further enhance pCR rates.

MK-2206 is an allosteric pan Akt (protein kinase B) inhibitor, with strong activity against isoforms 1 and 2, but is about 5-fold less potent for isoform 3 [13]. It interferes with the PI3K/mTOR axis thought to be essential for growth/survival signaling in multiple cancer types [14]. Because Akt sits at the nexus of such important growth and survival signaling pathways, and because we previously showed that Th1 cytokines also trigger apoptosis while lowering expression of HER family oncodriver expression [6], we hypothesized that the combination of Th1 cytokines and Akt antagonists might make a particularly effective pairing useful for immunotherapy of breast cancer. Since MK-2206 is one of the most potent and best-studied Akt agonists, we designed a series of studies around this agent to determine whether a phenotypically diverse panel of breast cancer lines would be susceptible to combined action of Th1 cytokines and MK-2206, whether this combination would enhance cell death through an apoptotic mechanism, and determine the effect of this treatment on the expression of important oncodrivers by breast cancer cells. Furthermore, we undertook to assess the impact Akt inhibition might have on critical cytokine production by T lymphocytes, and taking these effects into account, formulated in an *in vivo* mouse model of HER-2^pos^ disease immunotherapies based on either active vaccination or administration of recombinant cytokine to test whether these could pair with MK-2206 to enhance therapy. These studies can inform future clinical trials that pair immunotherapies with targeted small molecule inhibitor drugs.

## RESULTS

### MK-2206 and Th1 cytokines suppress metabolic activity in breast carcinoma lines

**Figure 1:**
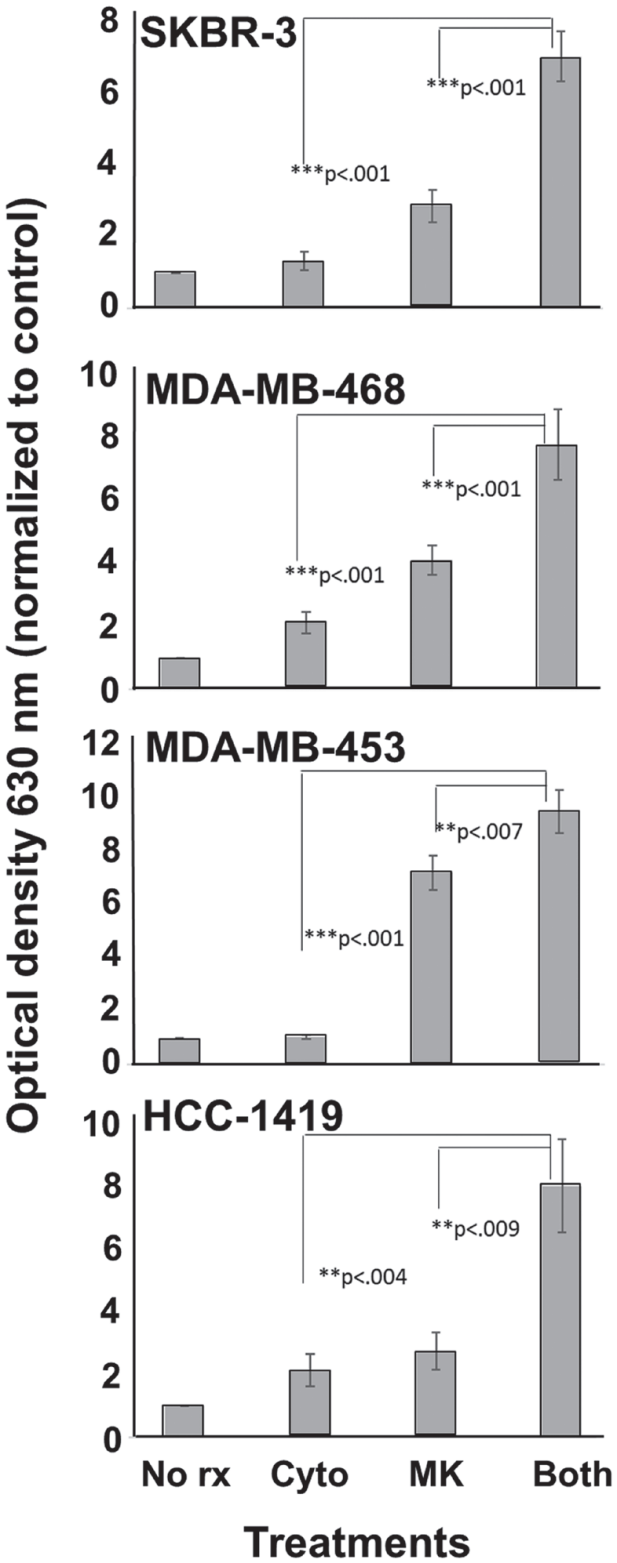
MK-2206 plus Th1 cytokines potentiate suppression of metabolic activity for human breast cancer cells. SKBR-3, MDA-MB-468, MDA-MB-453 and HCC-1419 cells were seeded at 5 × 10^3^ per well in 96-well cluster plate and cultured overnight. The cells were then exposed to Th1 cytokines (IFN-γ and TNF-α at 10 ng/ml each), MK-2206 (10 μM), both treatments, or left untreated, and incubated for an additional 72 h. Then, 20 μl of resazurin sodium salt solution (1.4 mg/ml) was added per well, and cells incubated until color change occurred. Optical density of culture supernatants was determined at 630 nm. Experiments were repeated at least three times for each cell line. Error bars depict the SEM.

We selected 4 human breast cancer cell lines for this study. These included SKBR3, MDA-MB-468, MDA-MB-453 and HCC1419. We began by performing dose-response studies, steadily increasing MK-2206 levels in the presence of a fixed concentration of Th1 cytokines (IFN-γ and TNF-α both at 10 ng/ml). Addition of Alamar Blue dye assessed cellular metabolic activity of treated cells. The redox dye works by being reduced through cellular oxidative metabolism with a subsequent loss of blue color. The loss is monitored spectrophotometrically so that lower OD values represent metabolically active cells, while high OD values mean the cells are not metabolically active and can’t alter the dye color. For all cell lines, the presence of Th1 cytokines lowered the concentration of MK-2206 necessary to cause substantive suppression of metabolic activity (Supplementary Figure 1). Based on the dose-response experiments, a concentration of 10 μM MK-2206 was selected for ongoing studies on all cell lines. Although there was line-to-line variation, 10 μM MK-2206 was a dose that gave relatively low activity as a single agent, but whose activity was strongly enhanced when cytokines were added. At this concentration, combined treatment with Th1 cytokines and MK-2206 resulted in significantly greater suppression of metabolic activity than either drug or cytokines alone for all tested cell lines (Figure 1). Isobolar analysis indicated that the activity of combined MK-2206 and Th1 cytokines was synergistic with calculated combinational indices for SKBR-3 cells (CI = 0.75), MDA-468 (CI = 0.11), MDA-MB-453 (CI = 0.53) and HCC-1419 (CI = 0.87) (Supplementary Figure 2).

### MK-2206 plus Th1 cytokines potentiate cell death

**Figure 2:**
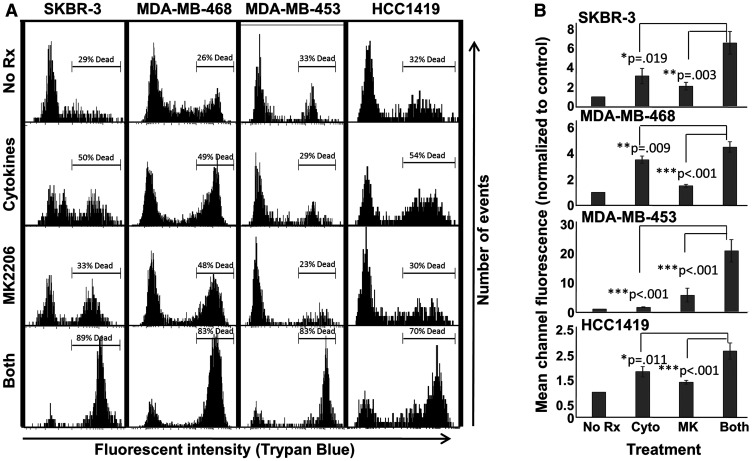
MK-2206 plus Th1 cytokines enhances cell death for breast cancer lines. SKBR-3, MDA-MB-468, MDA-MB-453 and HCC-1419 cells were seeded at 1 × 10^5^ per well in 12-well cluster plates and cultured overnight. The following day cells were then exposed to Th1 cytokines (IFN-γ and TNF-α at 10 ng/ml each), MK-2206 (10 μM), both treatments, or left untreated. After 72 h further incubation, cells were then harvested, washed and stained with Trypan Blue dye, then analyzed by flow cytometry for dye uptake. (**A**) Histogram analysis of a representative experiment. A region was defined to enumerate the percentage of dead (high-fluorescence, Trypan Blue-stained) cells. (**B**) Statistical analysis of composite data from at least 3 separate experiments per cell line using mean channel fluorescence. Error bars denote SEM.

Because suppressed metabolic activity can occur in the absence of actual cell death, we next subjected treated cells to Trypan Blue staining, which differentiates live from dead cells. Since Trypan Blue possesses fluorescent properties, staining can be monitored via flow cytometry [15, 16], with staining (dead) cells displaying a rightward shift in histograms (Figure 2A). We observed highest levels of cell death for all lines when cells were exposed to both MK-2206 and Th1 cytokines. Sometimes the combined effects were most dramatic; for example, with MDA-MB-453 cells, treatment with cytokines or MK-2206 only showed 29% and 23% dead cells, respectively. However, combined treatment increased cell death to 83%. We also analyzed the data from 3 separate experiments by mean channel fluorescence (Figure 2B), demonstrating that combined treatment led to significantly greater dye uptake (fluorescence) than single treatments for all tested cell lines.

### MK-2206 plus Th1 cytokines maximize markers of apoptosis

Cellular alterations occurring during apoptosis include mitochondrial membrane depolarization, the tendency to accumulate certain small molecule dyes in the nucleus, and membrane changes that expose phosphatidyl serine residues on the outer leaflet of plasma membranes. These alterations can be detected using TMRE, propidium iodide and fluorescently-labeled Annexin V, respectively.

**Figure 3:**
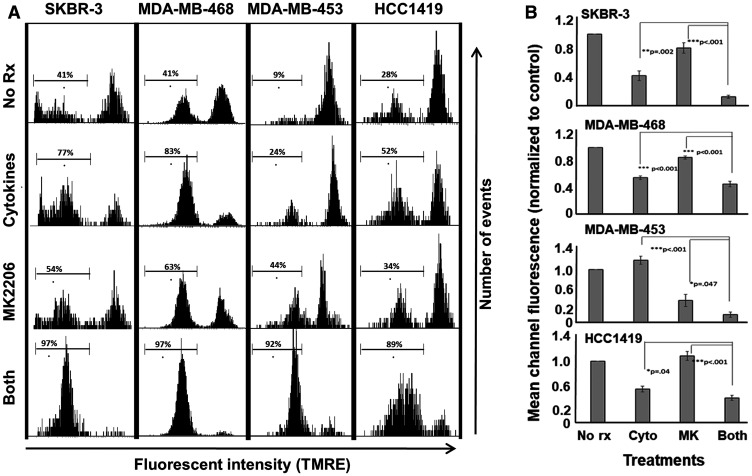
MK-2206 plus Th1 cytokines potentiate mitochondrial depolarization in human breast cancer lines. SKBR-3, MDA-MB-468, MDA-MB-453 and HCC-1419 cells were cultured at 1 × 10^5^ per well in 12-well cluster plates overnight. The next day cells were then exposed to Th1 cytokines (IFN-γ and TNF-α at 10 ng/ml each), MK-2206 (10 μM), both treatments, or left untreated. After 72 h further incubation, TMRE dye (final concentration 100 nM) was added to the cells which were incubated for an additional 20 minutes, after which cells were harvested, washed, resuspended in PBS and subjected to flow cytometry analysis. (**A**) Histogram analysis depicting a representative experiment. A region was defined to enumerate the percentage of low-staining (mitochondria-depolarized) cells. (**B**) Statistical analysis of composite data from at least 3 separate experiments using mean channel fluorescence. Error bars denote SEM.

TMRE mitochondrial depolarization assay showed that combined treatment with MK-2206 and Th1 cytokines induced the highest level of mitochondrial depolarization (leftward shift in histograms) for all four cell lines (Figure 3A) compared with single treatments. Composite data from three separate experiments analyzed by mean channel fluorescence likewise showed that combined treatment significantly reduced mitochondrial membrane potential compared to drug or cytokines alone (Figure 3B).

**Figure 4:**
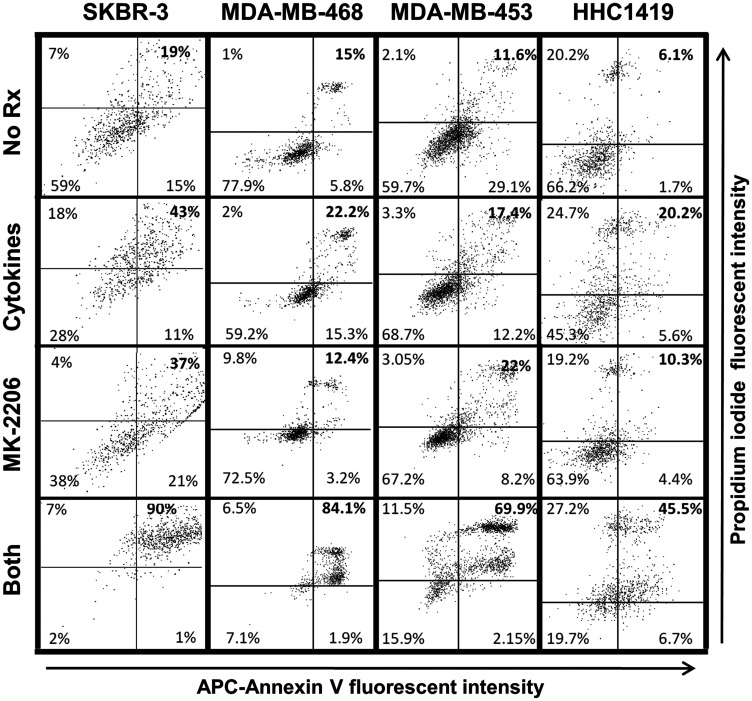
Combined MK-2206 and Th1 cytokines maximize markers of cellular apoptosis for human breast cancer cell lines. SKBR-3, MDA-MB-468, MDA-MB-453 and HCC-1419 cells were cultured at 1 × 10^5^ per well in 12-well cluster plates overnight. Cells were then exposed to Th1 cytokines (IFN-γ and TNF-α at 10ng/ml each), MK-2206 (10 μM), both treatments, or left untreated. After an additional 72 h incubation, cells were harvested, washed and resuspended in binding buffer, and then stained with propidium iodide (PI) and APC-labeled Annexin V. Stained cells were subjected to flow cytometry analysis. Quadrants were defined to differentiate live, double-negative cells (lower left quadrant) from apoptotic, double-positive cells (upper right quadrant), and establish percentages of cells within these categories. Data shown is one representative experiment from at least 3 individual trials for each cell line.

Annexin V/PI staining told a similar story. Cells in mid-apoptosis stain with both PI and labeled Annexin V. Dot-plot analysis revealed the percentage of double-staining cells for each treatment group: 19%, 43% 37% and 90% for untreated, cytokines, MK-2206 and both (SKBR3), 15%, 22.2%, 12.4% and 84.1%, respectively for MDA-MB-468; 11.6%, 17.4%, 22% and 69.9% respectively for MDA-MB-453 cells and 6.1%, 20.2%, 10.3% and 45.5%, respectively, for HCC-1419 (Figure 4). This experiment was repeated at least 3 times for each cell line, with similar results.

### Differential effect of combined MK-2206 plus Th1 cytokines and paclitaxel on breast cancer cells

**Figure 5:**
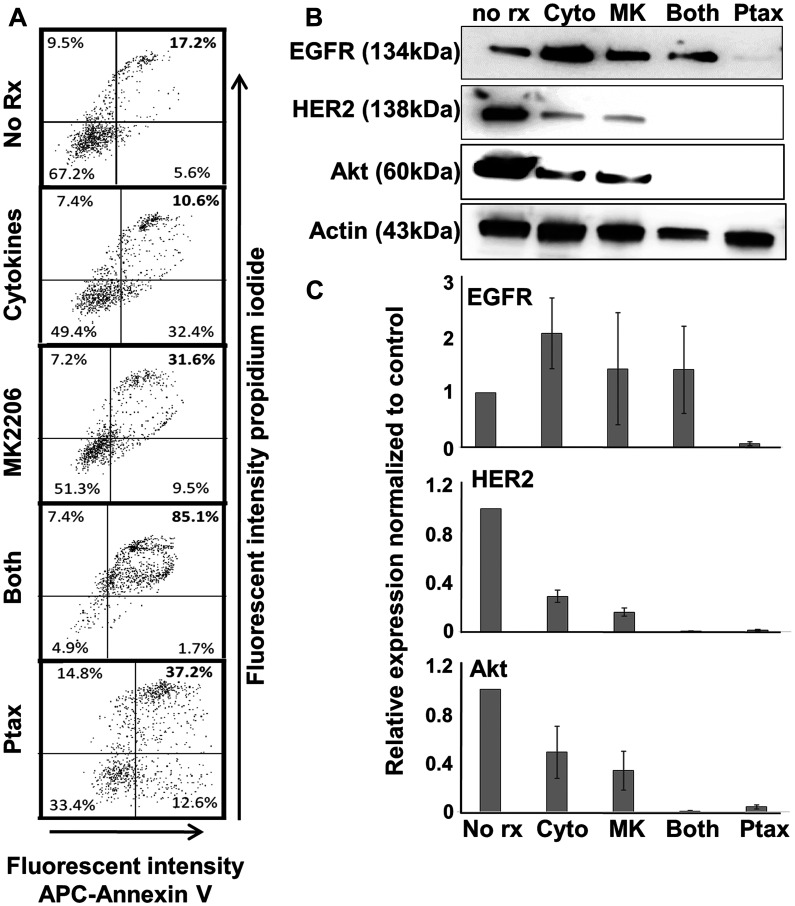
Th1 cytokines, MK-2206, and paclitaxel induce apoptosis in human breast cancer cells but differentially affect expression of oncodrivers. SKBR3 cells were seeded at a density of 1 × 10^5^ per well in 12-well cluster plates, and cultured overnight. The next day cells were exposed to Th1 cytokines (IFN-γ and TNF-α at 10 ng/ml each), MK-2206 (10 μM), both treatments, paclitaxel (300 nM) or left untreated. After 72 h further incubation, cells were harvested and (**A**) stained with propidium iodide and APC-labeled Annexin V. Stained cells were subjected to flow cytometry and data evaluated by quadrant analysis, with double-staining cells (upper right quadrant) defined as apoptotic. (**B**) Western blot analysis of cell extracts for expression of EGFR, HER2, Akt kinase and actin (loading control). (**C**) Densitometry analysis of western blots (composite data from 3 separate experiments) normalized to actin expression. Error bars indicate SEM.

Paclitaxel is a mainstay of breast cancer therapy. It stabilizes microtubules, interferes with orderly mitosis and promotes apoptosis [17, 18]. We wished to determine the relative capacity of this agent to induce apoptosis and other changes in breast cancer cells in comparison to MK-2206 plus Th1 cytokines. We initially performed dose-response studies to determine a dose of paclitaxel that induced maximal levels of apoptosis (Supplementary Figure 3), which was determined to be about 300 nM for the SKBR-3 cell line. We then compared this treatment to MK-2206, Th1 cytokines and both treatments via Annexin V/PI staining (Figure 5A). Under the conditions tested, cytokines alone did not enhance the proportion of double-staining events, while MK-2206 (31%) and paclitaxel (37%) each modestly enhanced the proportion of mid-apoptosis events over no treatment (17%). Combined treatment with cytokines and MK-2206, however, induced apoptosis in over 85% of cells. In addition, generally congruent combined treatment effects were observed for PARP and Caspase 3 cleavage as additional markers for apoptosis (Supplementary Figure 4).

Previous studies indicated that breast cancer cells, when undergoing Th1 cytokine-induced apoptosis, could down-regulate HER-2 [6]. We therefore assessed, via western blot analysis, the expression of EGFR, HER-2 and Akt in cells receiving various treatments (Figure 5B). As expected, Th1 cytokines partially suppressed HER-2 levels in SKBR3 cells. Interestingly, treatment with MK-2206 had a similar effect. However, MK-2206 plus Th1 cytokines diminished HER-2 levels virtually to the point of non-detection. Surprisingly, despite paclitaxel’s lower levels of induced apoptosis, this drug alone profoundly reduced HER-2 levels to nearly undetectable levels. Also of interest were changes in EGFR expression. It appeared that neither cytokines nor MK-2206 suppress EGFR expression, but instead somewhat increased levels in cells. Paclitaxel, on the other hand strongly suppressed EGFR expression, to a level similar to HER-2. Akt followed the pattern of HER-2, with either Th1 cytokines or MK-2206 providing partial suppression, while combined treatment and paclitaxel alone leading to near total elimination of Akt.

### Antagonism of Atk pathway leads to suppressed IFN-γ production by lymphocytes

**Figure 6:**
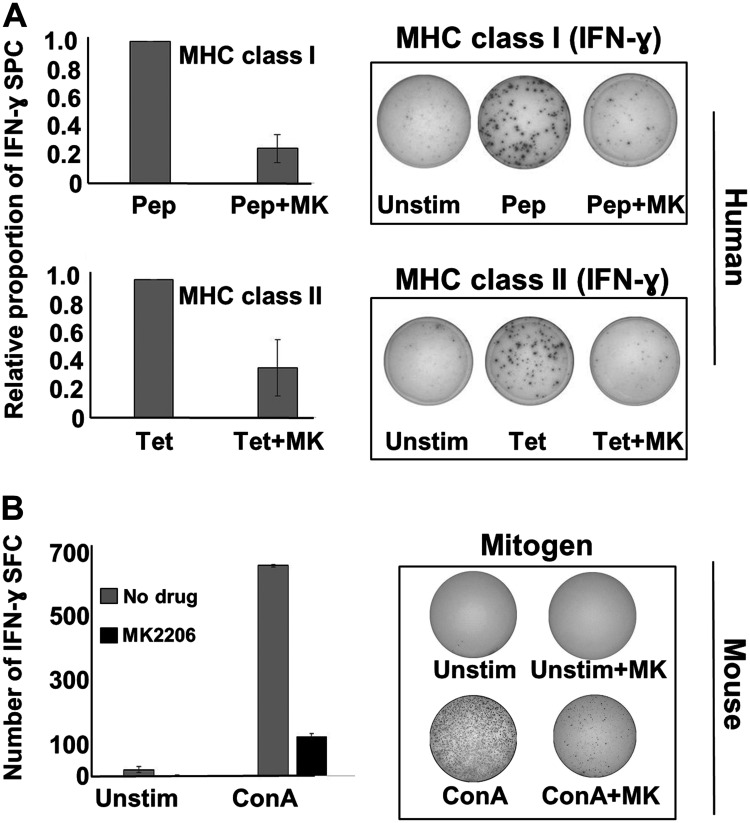
MK-2206 suppresses IFN-γ responses in human PBMCs and murine splenocytes. (**A**) Cryopreserved human PBMCs from healthy donors were thawed and plated at a density of 3 × 10^5^ per well in human IFN-γ 96-well Elispot plates. Cells were then stimulated with either a mixture of peptides based on the sequence of common viral antigens (upper panels) or tetanus toxoid (lower panels) in the presence or absence of MK2206 (10 μM). Elispot plates were incubated overnight, then developed and IFN-γ spot forming cells enumerated. Data expressed as the percentage of the positive control (left panels), with photographs of representative, developed wells also shown (right panels). (**B**) Freshly prepared mouse splenocytes were cultured in IFN-γ elispot plates at 3 × 10^5^ per well and then exposed to mitogen (concanavilin A) in the presence or absence of MK2206 (10 μM). Cells were incubated overnight and the next morning plates were developed and IFN-γ spot forming cells enumerated. Left panel represent total IFN-γ spots obtained with various treatments, with error bars denoting SEM from triplicate wells. Right panels show photographs of representative developed wells.

Our overarching goal of these studies was to test a treatment incorporating a small molecule inhibitor drug like MK-2206 and an immunotherapy, such as the Th1-polarizing DC-based immunization platform we have developed. However, because of the ubiquitous nature of Akt, we were concerned that blocking Akt action through MK-2206 over other drugs could have off-target effects on T cells that would mitigate its effectiveness as a partner with vaccination or other immunotherapy. To test this hypothesis, we performed some standard assays of T cell activation and function, using the production of IFN-γ as a readout for activity, as assessed via the Elispot assay (Figure 6). Human PBMCs were therefore stimulated either with a pool of MHC class I-binding peptides based on the sequence of several common viral antigens, or with tetanus toxoid (recall antigens for CD8^pos^ and CD4^pos^ T cells, respectively), either in the presence or absence of MK-2206. For stimulation using MHC class I-binding viral peptides, there was a marked diminution of IFN-γ spot-forming cells (Figure 6A upper panels) in the presence of MK-2206. Likewise, stimulation with whole tetanus toxoid in the presence of MK-2206 also resulted in dramatically fewer IFN-γ spot forming cells (Figure 6A lower panels). Similarly, we also stimulated murine splenocytes with mitogen in the presence or absence of MK-2206 and again observed a strong suppression of IFN-γ responses (Figure 6B).

**Figure 7:**
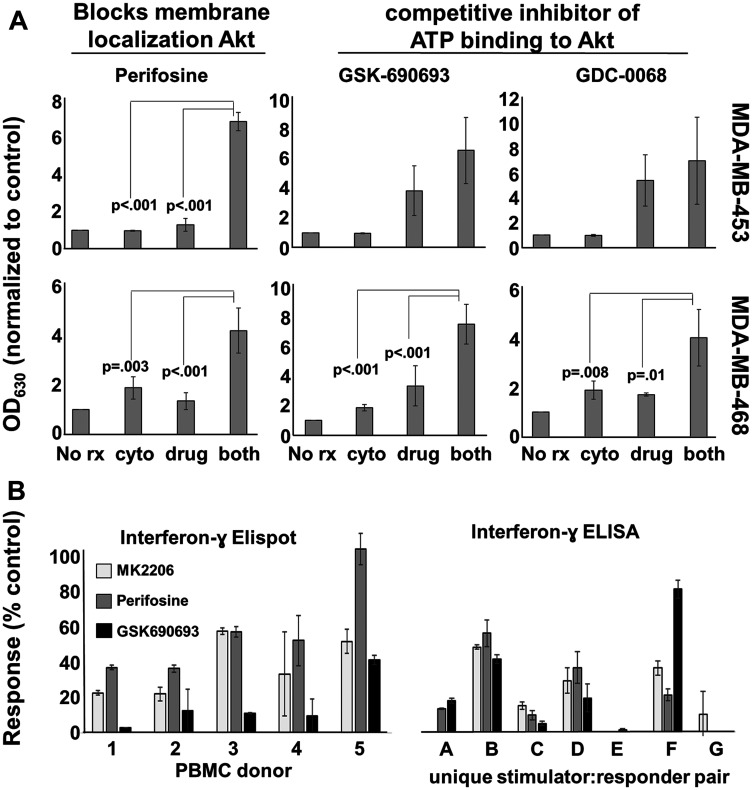
Other Akt antagonists display similar apoptosis-inducing and T-cell inhibitory properties as MK-2206. (**A**) MDA-MB-453 and MDA-MB-468 cells were cultured overnight in 96-well plates. The next day they were treated with perifosine (20 μM), GSK-690693 (10 μM), or GDC-068 (40 μM) in the presence or absence of Th1 cytokines (IFN-γ and TNFα at 10ng/ml each). After 72 hours of treatment, 20 μl resazurin sodium salt solution (1.4 mg/ml) was added to each well and the cells were incubated until color change occurred. Optical densities were read spectrophotometrically at 630 nM. Shown are composite data from at least three trials per cell line expressed as mean optical density +/– SEM. (**B**) An IFN-γ ELISPOT was performed according to manufacturers’ instructions using PBMCs from 5 different donors (left panel). The PBMCs were cultured with viral recall peptides overnight in the presence of MK2206 (10 μM), Perifosine (20 μM), GSK690693 (10 μM), or left untreated for control. The next day the number of spot forming cells were counted and the data expressed as the percent of the control. IFN-γ ELISA analysis of 48-hour culture supernatants from allogeneic MLRs where activated dendritic cells (DC) and lymphocyte-rich elutriation fractions were co-cultured at 1:40 stimulator: responder ratios in the presence or absence of drug. Data displayed represents the percent maximum of mean IFN-γ production from seven unique allogeneic DC: lymphocyte pairings.

There were at least two possible explanations for these observations. First, MK-2206 was blocking targets in addition to Akt, and these off-target molecule activities were responsible for T cell suppression. Second, blocking Akt activity itself was destructive to T cell function. To distinguish between these two possibilities, we first verified that additional, structurally-unrelated compounds (that block Akt activity by different mechanisms) also demonstrated similar anti-breast cancer cell activity as MK-2206 when combined with Th1 cytokines. We then tested these other compounds for their ability to interfere with IFN-γ production by stimulated lymphocytes. We selected three compounds for testing: perifosine, which inhibits Akt activity through inhibiting its interaction with PIP, as well as GSK-690693 and GDC-0068, which are both competitive inhibitors of Akt, acting on the ATP binding pocket of the enzyme [19]. We tested these compounds against two cancer lines, MDA-MB-468 and MDA-MB-453 cells. In the Alamar Blue assay, the combination of perifosine and Th1 cytokines induced significantly greater metabolic inhibition in both tested cell lines than either drug or cytokine treatment alone (Figure 7A left panels). For GSK-690693 and GDC-0068, statistical significance for combined treatment versus single treatments was observed for MDA-MB-468 cells, but this same trend observed for MDA-MD-453 cells did not reach actual statistical significance (Figure 7A center and right panels). Nonetheless, the same general pattern was observed for these drugs as we had seen previously for MK-2206, suggesting that a feature of any drug that strongly blocked Akt activity would be its ability to combine well with Th1 cytokines to enhance anti-cancer cell activity.

These additional Akt-antagonist drugs were then tested for their capacity to interfere with T cell activity. We did this two ways; via Elispot analysis, and through the use of the allogenic MLR. For Elispot analysis, PBMCs from 5 donors were stimulated with MHC class I-binding viral peptide pool in the presence or absence of perifosine, GSK-690-693 or GDC-0068, and cultures assessed for their ability to demonstrate IFN-γ spot forming cells (Figure 7B left panel). For the allogenic MLR, cells from monocyte-rich elutriation fractions of one donor (stimulator cells) were co-cultured with cells from lymphocyte-rich elutriation fractions obtained from different donors (responder cells), and 72 hour supernatants collected and assessed for the presence of IFN-γ using ELISA analysis (Figure 7B right panel). Despite an unexpected level of person-to-person variability in the magnitude of suppression, it was nonetheless clear that all three drugs had a tendency to strongly interfere with IFN-γ production by stimulated cells. It thus appeared that any drug that antagonized Akt activity was likely to interfere with elements of T cell function, and thus could present difficulties for combining Akt-blocking drugs with active immunotherapy that relied on at least IFN-γ production by T cells.

### Recombinant interferon gamma plus MK-2206 suppresses tumor growth *in vivo*

**Figure 8:**
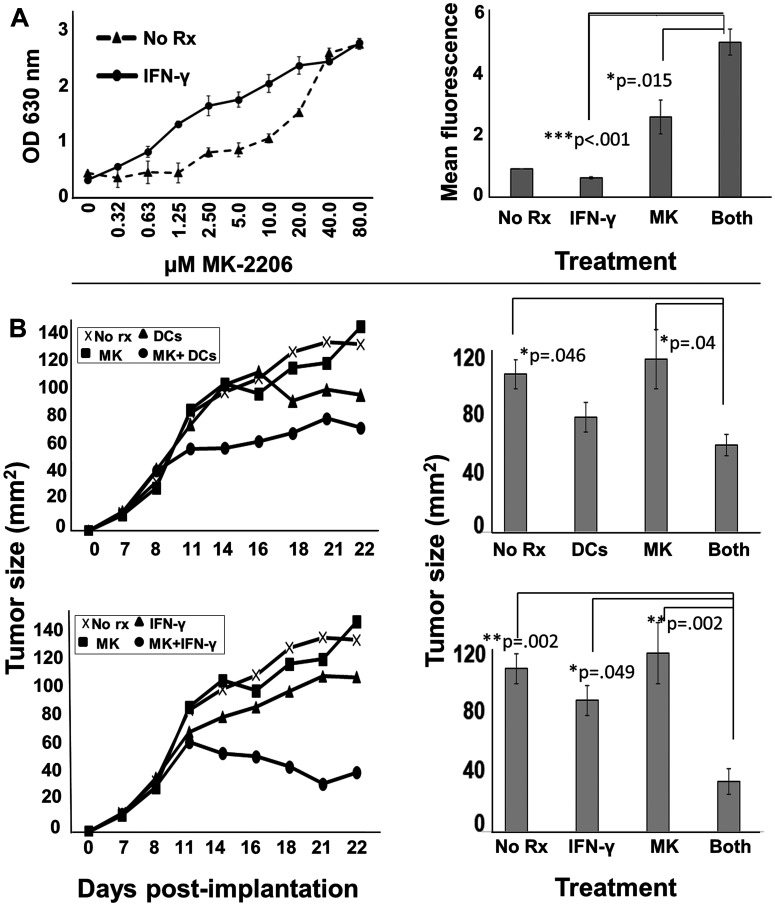
MK-2206 in conjunction with immunotherapy slows progression of rodent HER-2^pos^ tumors. (**A**) Erbb2^pos^ (rat homolog of HER-2) TUBO cells were cultured overnight in 96-well culture plates. The next day they were exposed to increasing concentrations of MK2206 in the presence (solid circles) or absence (dotted triangles) of IFN-γ (50 ng/ml) and incubated a further 72 h, after which 20 μl of resazurin sodium salt solution (1.4 mg/ml) was added to each well, and upon color change, optical density of culture supernatants assessed at 630 nm (left panel). Cells similarly treated were also assessed by Trypan Blue staining, and dye uptake evaluated by flow cytometry (right panel). Error bars indicate SEM. (**B**) 5 × 10^5^ TUBO cells were implanted into the fat pad of one of the right mammary glands of female Balb/c mice. When tumors became palpable (seven days), mice were provided with six treatment regimens (5 mice per group) including no treatment (upper and lower panels), MK2206 (upper and lower panels), rat HER-2 peptide-pulsed dendritic cells (upper panels), peptide-pulsed DCs plus MK2206 (upper panels), IFN-γ (lower panels) and IFN-γ plus MK2206 (lower panel). MK2206 was supplied in two 5-day cycles separated by a 2 day rest period. IFN-γ was supplied on the same schedule as MK-2206. DCs were administered twice weekly during cycles of MK2206 treatment. Tumor growth was monitored 2–3 times per week. Growth curves are displayed in left panels, and statistical analysis at the terminus of treatment displayed in right panels. Error bars indicate SEM.

Because MK-2206 suppresses IFN-γ production, it could interfere with the effectiveness of a therapeutic vaccine. However, IFN-γ, unlike TNF-α, can be safely supplied systemically in recombinant form to humans. Therefore, it is possible that a parenterally-administered cytokine could substitute for vaccination, provided IFN-γ could enhance the effect of MK-2206 even in the absence of TNF-α. Prior to testing a murine therapy model, we verified *in vitro* that IFN-γ alone could enhance MK-2206 activity on the relatively difficult-to-kill HCC-1419 cells (Supplementary Figure 5). We found that the fraction of cells undergoing apoptosis in cells treated with MK-2206 plus IFN-γ was 47%, almost identical to the 45.5% observed previously when both TNF-α and IFN-γ were paired with drug (Figure 4). Similarly, we performed a MK-2206 dose-response study on TUBO murine HER-2^pos^ breast carcinoma cells in the presence or absence of murine IFN-γ (Figure 8A, left panel). Although IFN-γ by itself had little effect on TUBO cells, its presence decreased, by several fold, the concentration of MK-2206 necessary to cause a comparable level of metabolic suppression in the Alamar Blue assay. Likewise, Trypan Blue staining demonstrated that the dual treatment of IFN-γ plus MK-2206 causes a statistically significant increase in dye uptake compared with single treatments (Figure 8A right panel).

Finally, we tested *in vivo* two immune-based therapies (DC-based vaccination and recombinant IFN-γ) in conjunction with MK2206. TUBO cells were implanted into the region of fat pad of the mammary gland. After tumors became palpable (7 days post-implantation), mice were divided into six treatment groups: untreated, MK-2206 alone, peptide-pulsed DC vaccine alone, IFN-γ alone, drug plus vaccine or drug plus cytokine. Both MK-2206 and cytokine was given in two 5-day cycles separated by a two-day rest. Vaccines were given twice weekly over the same 2 weeks. We found that neither MK-2206 nor IFN-γ as single agents significantly suppressed tumor growth compared with the vehicle group (Figure 8B). DC vaccination alone appeared to show some slowing of tumor growth, but it was not statistically significant (Figure 8B upper left and upper right panels). In contrast, DC vaccination plus MK-2206 significantly delayed tumor progression compared with no treatment group. Likewise, recombinant IFN-γ plus MK-2206 delayed tumor growth (*p* = .002), and appeared to be the most effective therapy tested (Figure 8B lower left and right panels).

## DISCUSSION

The success of STI-571 (gleevec/imatinib), an inhibitor of the Bcr/Abl fusion oncogene, not only transformed the treatment of chronic myelogenous leukemia (CML) [20], but it also spurred an explosion in the development of additional small molecule inhibitors directed against various other oncodrivers. Unfortunately, despite billions of dollars invested, none of these agents have met with the same kind of dramatic success in solid tumors that gleevec showed for CML. Nonetheless, a number of targeted agents have demonstrated enough clinical activity to gain FDA approval (with others in the pipeline), and are now used routinely in the clinic; the caveat being they usually have only marginal activity as single agents, and must therefore be combined with other, more traditional therapies to observe real gains in outcomes. Notwithstanding the approval and adoption of a number of such targeted small molecule inhibitors, it is safe to say that the actual utility of most of these agents has yet to live up to their initial high expectations. Given the heavy investment in developing small molecule inhibitor drugs, both in dollars and in man-hours of research, it is reasonable to explore all avenues to obtain their maximal therapeutic potential. Indeed, acknowledging these investments in currently-available drugs, the notion of “drug repurposing and repositioning” has been advanced, which advocates finding new uses for approved drugs, or finding optimized settings for these existing drugs [21]. One way to implement this recommended approach is to combine such drugs with immunotherapeutic strategies, including the DC-based immunization platform developed in our laboratory. We used such an approach to improve complete response rates by nearly 6-fold in HER2^pos^/ER^pos^ early breast cancer by combining a brief course of anti-estrogen therapy with anti-HER-2 Th1-polarizing vaccination [9].

Akt kinase (protein kinase B) is a serine-threonine kinase that exists in 3 isoforms. Akt sits at the nexus of important pro-growth and pro-survival pathways [22]. Akt gains activation usually when various membrane receptor tyrosine kinases are engaged by their ligands or alternatively through activity of G-protein coupled receptors. This leads to the activation of phosphotidylinosidite 3-phosphate kinase (PI3K), which in turn converts the membrane component phosphatidylinositol bisphosphate (PIP2) into phosphatidylinositol triphosphate (PIP3). PIP3 interacts with special domains on Akt [23], allowing its association with the plasma membrane, a location required for its activity. There it is phosphorylated [24], a process through which full enzymatic function is finally imparted. Although Akt has numerous substrates in multiple diverse signaling pathways, its overall activity is generally considered to be promoting of growth, division and survival, while simultaneously inhibiting pro-apoptotic pathways [25]. Its composite activity is thus considered favorable to oncogenesis. This makes Akt an attractive target for the development of novel anti-cancer agents [26].

A number of anti-cancer agents have in fact been developed that target Akt, and these can be classified as allosteric inhibitors, ATP competitive inhibitors, and irreversible inhibitors. MK-2206 is a representative of the allosteric inhibitors. The first trials with this compound (as a single agent) seemed to show acceptable tolerability and demonstrable *in vivo* suppression of Akt activity [27, 28]. There were associated toxicities observed, however, including GI symptoms, skin rash, fatigue and hyperglycemia. Other phase I studies combined MK-2206 with additional drugs such as carboplatin/paclitaxel, docetaxel, erlotinib, dalotuzumab, MK-0752 and selumetinib and anti-estrogen therapy [29–32]. Early combination therapies hinted at the potential for favorable clinical activity [33–36]. Other studies led to findings that were less hopeful. A phase II monotherapy study in patients with advanced breast cancers with PIK3CA or Akt mutations showed very limited clinical activity [37]. Likewise, completion of the trial testing MK-2206 plus anti-estrogen therapy for ER^pos^ breast cancer failed to show clinical activity above that of anastrazole alone [38]. Finally, a trial using MK-2206 as a monotherapy prior to scheduled surgical resection of operable breast cancer was stopped due to unexpectedly high toxicity, given this particular clinical setting [39]. It therefore appears that MK-2206, like other Akt inhibitors currently being tested, are of unproven value therapeutically, at least as part of the therapy regimens tested up to this point.

We found that among the four human breast cancer lines tested, the combination of MK-2206 and Th1 cytokines (IFN-γ and TNF-α) caused greatly enhanced cell death compared with either treatments alone, and this death occurred through an apparent apoptotic mechanism. We had shown in previous studies that Th1 cytokines could cause a down-regulation of HER-2 and HER-3 in some breast cancer lines (while expression of other proteins like EpCAM were unperturbed), and this suppression, at least for HER-2, could be enhanced by the addition of the small molecule inhibitor of HER-2 and EGFR, lapatinib [6, 16]. We observed a similar effect here with MK-2206; Either drug or Th1 cytokines alone lowered HER-2 expression in treated cells, but both together brought the proteins down to nearly undetectable levels. The same was also observed for total Akt levels. Interestingly, there were differences in the expression of pro-growth and proliferation proteins in cells treated with cytokines plus MK-2206 and those treated with paclitaxel, a first-line anticancer agent that also induces apoptosis in breast cancer cells [17]. Despite the fact that optimized concentrations of paclitaxel did not cause as high a percentage of apoptotic cells as MK-2206 plus Th1 cytokines (30% versus 85%; see Figure 5), it nonetheless virtually eliminated EGFR expression in treated cells. In contrast, there was little apparent difference between EGFR levels in untreated cells verses those treated with cytokines plus MK-2206. In fact, Th1 cytokines alone appeared to somewhat enhance EGFR expression. On the other hand paclitaxel, like the combination of MK-2206 and Th1 cytokines, strongly suppressed both HER-2 and Akt levels. It is likely that cells undergoing apoptosis selectively down-regulate pro-growth and pro-survival gene products, so that signaling through these do not antagonize the process of apoptosis, once cells are fully committed. These findings also suggests, because of the differences in EGFR expression, that the apoptotic processes induced by Th1 cytokines, with and without added MK-2206, is somehow biochemically distinct from that induced by paclitaxel.

Few studies have examined the impact of Akt antagonist drugs on T cell function. One recent study did demonstrate that 48h exposure of normal unstimulated T lymphocytes to MK-2206 showed very limited loss of viability to these resting cells (about 10% loss). In contrast, T cells treated with mitogen in the presence of MK-2206 were more sensitive, with recorded viabilities at a bit less than 70% compared with controls [40]. Thus cellular activation in the presence of Akt inhibition appeared somewhat toxic to T cells. Functional analysis, however, was not performed in these studies. In contrast, we focused our investigation on suppression on T cell function, namely the capacity of antigen- and mitogen-stimulated cells to produce IFN-γ. We showed that MK-2206, as well as all other tested Akt antagonists, strongly suppressed IFN-γ production by stimulated lymphocytes. From our standpoint, the capacity to produce this archetypical Th1 cytokine is one of the most important aspect of T cell activity. Previous studies by others have shown that Th1-type T cells are associated with superior outcomes for breast cancer [41]. It is entirely possible that one of the reasons Akt inhibitor drugs have underperformed expectations clinically is that they simultaneously suppress activated T cells, and particularly their capacity to produce cytokines like IFN-γ which might be critical for anti-tumor activity by the immune system. Interestingly, pairing DC vaccination in a mouse model with administration of MK-2206 seemed to provide only relatively weak enhancement of activity compared with DC vaccination alone, perhaps due to interference of MK-2206 with vaccine-induced effectors of immunity. Such interference was documented previously with the drug sunitinib, which could be mitigated through staggering drug and vaccine therapy [42]. We chose a different strategy to circumvent the possibility of such interference by supplying recombinant IFN-γ concurrent with MK-2206. This approach resulted in statistically significant and much stronger suppression of tumor outgrowth compared with single treatments. This seems to present a clinically translatable strategy to enhance the effects of Akt antagonist drugs. IFN-γ is already a standard of care treatment for chronic granulomatous disease [43] and also is indicated for some forms of osteopetrosis. Interestingly, the combination of IFN-γ and anti-HER2 antibodies (which exert anti-tumor activity via blocking growth factor signaling as well as through other mechanisms) has shown anti-tumor activity in mouse models [44]. It is also an approach being currently tested in a clinical trial (NCT03112590) with locally advanced HER-2^pos^ breast cancer. For these patients, the standard of care typically includes two anti-HER-2 antibodies (trastuzumab and pertuzumab) plus carboplatin and a taxane like paclitaxel. The experimental therapy of this trial substitutes IFN-γ for carboplatin. Although still ongoing, the results are encouraging, with patients demonstrating nearly double the pCR rates compared with what would be expected for the standard of care regime, even while eliminating its harshest component (i. e. the carboplatin). In light of this developing clinical trial, in the present study we placed particular emphasis on trastuzumab- and lapatinib-resistant cell lines, positioning Akt antagonist drugs as possible ways to deal with resistance to these more established drugs, should they come to be used more routinely in conjunction with IFN-γ. It thus appears that combining this cytokine with therapies comprised at least in part of targeted agents may represent a generalizable approach for improving outcomes, and one that probably warrants continued exploration.

## MATERIALS AND METHODS

### Cell culture

HER-2^pos^ SKBR-3, triple negative MDA-MD-468, HER-2^pos^, trastuzumab resistant HCC1419, and HER-2^pos^, trastuzumab and lapatinib resistant MDA-MB-453 cell lines were obtained from American Type Culture Collection (ATCC; Rockwell, MA). MDA-MB-468, MDA-MB-453 and HCC1419 cell lines were re-authenticated by ATCC using STR prior to submission. Assays preformed using the SKBR-3 cell line were done within 6 months after the time of purchase from ATCC. Murine TUBO cells were a kind gift from Dr. Wei Zen Wei, (Wayne State University) and were derived from a neu transgenic mouse [45] and were cultured in RPMI medium. SKBR-3 cells were cultured in McCoy’s 5A Media (Corning, Manassas, VA), MDA-MB-468, MDA-MB-453, and HCC1419 cells were cultured in RPMI-1640 (ATCC). All media was supplemented with 10% v/v fetal calf serum (FBS; Atlanta Biologicals, Flowery Branch, GA), 100 units/ml of penicillin and 100 μg/ml of streptomycin sulfate (BioWhittaker, Walkersville, MD), 2 mmol/L glutamine (BioWhittaker), 1 mmol/L sodium pyruvate (BioWhittaker), and 1% non-essential amino acids (BioWhittaker). All cells were maintained in culture at 37°C in 5% CO_2_ in a humidified atmosphere.

### Alamar blue assay

The Alamar Blue assay was used as previously described [16]. Briefly, breast cancer cell lines were cultured in 96-well cluster plates and treated the next day with 20 ng/ml TNF-α and 10 ng/ml of IFN-γ, and varying concentrations of MK-2206 (Selleckchem, Houston, TX), or paclitaxel (Selleckchem) and incubated for approximately 72 hours. On day 3, resazurin sodium salt was added to each well and plates were further incubated until color change of dye was observed. The reduction of resazurin to resorufin was measured via optical density at 630 nm with a Bio-Tek ELx800 absorbance reader running Gen5 analysis software.

### Trypan blue exclusion assay

Cells were plated and treated in a manner identical to that described for the Alamar Blue assay. On day 3, as previously described [16] cells were harvested, washed by centrifugation, and resuspended in a 0.002% (w/v) solution of trypan blue in PBS. Cells were analyzed via an Amnis/Millipore FlowSight flow cytometer using an excitation wavelength of 642 nm and emission detection between 642 nm and 740 nm.

### Annexin V/Propidium iodide apoptosis assay

Cellular apoptosis was assessed via staining with fluorescently-labeled Annexin V and propidium iodide (PI) followed by flow cytometry analysis as similarly described [16]. Cells were treated in a manner identical to that described for the Alamar Blue assay with the exception of being plated at a density of 1 × 10^5^ cells per well in 12-well cluster plates. On day 3, the cells were harvested, washed via centrifugation, and resuspended in 50 μl annexin-binding buffer (Invitrogen, Eugene, OR)). To the cell suspension, 5 μl of APC-conjugated Annexin V (BioLegend, San Diego, CA) was added and allowed to incubate for 15 minutes at room temperature in the dark. Cells were then washed and resuspended in 50 μl of annexin-binding buffer. Prior to analysis, 10 μl of 100 μg/ml PI (Invitrogen) was added to the cell suspension and incubated for 10 minutes. Annexin V binding and PI uptake was assessed with a FlowSight flow cytometer. APC fluorochrome was excited using a 642 nm laser with emission detection between 642 and 740 nm. Propidium iodide was excited with a 642 nm laser and emission detected between 642 and 740 nm.

### Mitochondrial membrane potential assessment (TMRE)

As previously described [16], cells were incubated for 30 minutes with 100 nM TMRE (tetramethylrhodamine, ethyl ester) (Sigma, St. Louis, MO) before being centrifuged, washed once with PBS, resuspended in 40 ul of PBS, and transferred to microcentrifuge tubes for FACS analysis. The cells were then analyzed via FACS (Flowsight, Amnis-Millipore) using a 488 nm excitation laser. Intact and single cells were gated and the mean channel fluorescence of the gated cells was used to assess cells with active mitochondria.

### Western blotting

Cells were plated and treated as described above. On day 3, similar to previously described [16], culture media and cells were harvested, pelleted via centrifugation, and washed twice with ice-cold PBS, and 100 μl of RIPA buffer containing protease inhibitor cocktail (Pierce, Rockford, IL) and PhosStop phosphatase inhibitor cocktail (Roche, Mannheim, Germany) was added. Lysates were placed in tubes on ice for 30 min, with intermittent vortexing. Lysates were centrifuged at 13,000g for 20 minutes at 4°C and supernatants were collected. A Bradford protein assay was performed to determine total protein concentration so that 50 μg of total protein could be loaded into each well of a 4–15% Mini Protean TGX gel (Bio-Rad, Hercules, CA). The gels were run and transferred onto a PVDF membrane (Bio-Rad). Membranes were blocked with 1% BSA in TBS-T for 1 hour and then incubated with primary antibodies in blocking buffer (1:1000) overnight at 4°C. Membranes were then washed and incubated with HRP-conjugated secondary antibodies in blocking buffer (1:10,000) for 1 hour at room temperature. Membranes were then washed and bound antibody was detected via SuperSignal^®^ West Pico Chemiluminescent Substrate (Thermo Scientific) and visualized using an ImageQuant LAS 4000 mini (GE Healthcare). Membranes were washed and re-probed for β-actin as a loading control. Analysis of densitometry was done with Image J and normalized to β-actin loading control. Antibodies Used: anti-EGFR (H11), Thermo Scientific; anti-HER-2/ErbB2 (PA5-14632), Thermo Scientific; anti-Akt1 (B-1), Santa Cruz Biotechnology; anti-Actin (C4), EMD Millipore; anti-mouse-IgG_k_ BP-HRP and goat anti-rabbit IgG-HRP, both Santa Cruz.

### Lymphocyte functional assays

For allogeneic MLRs (as previously described [16]), human peripheral blood mononuclear cells were obtained (after provision of informed consent) from healthy volunteers via leukapheresis, and in accordance with the principals of the Declaration of Helsinki and NIH guidelines for human subjects, through protocols approved by the Institutional Review Boards of the Cleveland Clinic (08–957) and Kent State University (18–421). Blood products were separated into either CD14^pos^ peripheral blood monocyte- or lymphocyte-enriched fractions via countercurrent centrifugal elutriation as described previously [46] and maintained cryopreserved in liquid nitrogen until use. Dendritic cells were derived from monocytes that were thawed and incubated overnight in Macrophage SFM media (Gibco) supplemented with GM-CSF (50 ng/ml) and IL-4 (1000U). The next morning 1000U of IFN-γ was added to the culture and after 2 additional hours LPS (50 ng/ml) was added. The cells were allowed to incubate for another 4 hours before being harvested, washed, and resuspended in RPMI supplemented with 5% human AB serum. Allogeneic lymphocyte-enriched fractions were thawed and brought up in identical culture medium. The cells were combined at a ratio of 20:1 (lymphocytes: dendritic cells) and plated in a 48-well cluster dish at 1ml total volume per well. The allogeneic co-culture was either treated with MK-2206 (10 μM) or left untreated for control. After 72 hours the supernatants were collected and analyzed for IFN-γ via ELISA, and the cells were harvested and stained with FITC- anti-CD4 antibody (Biolegend) and APC-anti-CD69 antibody (Biolegend). Surface expression was assessed via Flow cytometry using the Flowsight (Amnis-Millipore).

ELISPOT was performed similarly to our previous report [16]. Briefly, cryopreserved, unfractionated total peripheral blood mononuclear cells (PBMCs) and IFN-γ ELISPOT kits were purchased from Cellular Technology Limited (C. T. L., Shaker Heights, OH). As per manufacturer’s recommendations, the PBMC’s were thawed and plated at a density of 200,000 cells per well (either in the presence or absence of 10 μM MK-2206) with CEF-Class I Peptide Pool “Plus” (a mixture of common viral peptides; 10 μl/well, C. T. L) or tetanus toxin (2 μg/well, Sigma) serving as recall antigens, or incubated in media alone (control). The next day the cells were removed and the number of IFN-γ spot-forming cells were assessed as per kit instructions. All plates were read on an ImmunoSpot analyzer (C. T. L).

### Generation of mouse dendritic cells

Immature dendritic cells (DCs) were obtained from the bone marrow of female Balb/c mice as previously described (Cohen STAT paper). Briefly, bone marrow was harvested from Balb/c mouse femur and tibia, cultured at 5 × 10^5^/ml for 6 days in culture RPMI (RPMI containing 10% FBS, 2 mM glutamine, .1 mM nonessential amino acids, 100 units/ml sodium pyruvate, and 100 mg/ml penicillin/streptomycin) supplemented with 30 ng/ml human Flt-3L and 25 ng/ml murine IL-6 (both from Peprotech, Rocky Hill, NJ). On day 6 cells were harvested, washed 2 times in PBS, resuspended at 2 × 10^6^/ml in culture RPMI supplemented with 50 ng/ml murine GM-CSF and 10 ng/ml of murine IL-4 (both from Peprotech), and incubated overnight. The next day the cells were harvested and frozen down at 2 × 10^7^ in FBS containing 10% DMSO. On day of vaccination cells were thawed, washed once to remove DMSO, and then resuspend at 2 × 10^6^ in culture RPMI containing only 1% FBS and supplemented with murine GM-CSF (50 ng/ml) and IL-4 (10 ng/ml). After 1 hour of incubation the cells were exposed to murine HER-2 peptides (2 class II and 1 class I), incubated for 2 additional hours, activated with 20 ng/ml LPS and 10 ng/ml CpG (Invivogen, San Diego, CA), incubated for 2 more hours, harvested, washed 3 times in PBS, and resuspended in PBS at a concentration of 1 × 10^7^/ml for vaccination.

### Therapy model

Female Balb/c mice, 50–56 days old, were purchased from Charles River Laboratories (Wilmington, MA). They were maintained in a specific pathogen-free environment in accordance with the National Institute of Health’s guidelines. Experiments were approved by the Institutional Animal Car and Use Committee (IACUC) of Kent State University under protocols 451-GK-17-15 and 481 GK-19-05. The TUBO cell line was generated previously from a spontaneous tumor in a Balb-neuT mouse, a transgenic line that overexpresses rat HER2 (neu) driven by the mouse mammary tumor virus promoter [45]. TUBO cells were cultured in RPMI supplemented with 10% v/v fetal calf serum (FBS; Atlanta Biologicals, Flowery Branch, GA), 100 units/ml of penicillin and 100 μg/ml of streptomycin sulfate (BioWhittaker, Walkersville, MD), 2 mmol/L glutamine (BioWhittaker), 1 mmol/L sodium pyruvate (BioWhittaker), and 1% non-essential amino acids (BioWhittaker). Cells were harvested with Cell Dissociation Buffer (Gibco, Grand Island, NY), washed 2 times with PBS, and then resuspended in PBS for inoculation.

Mice were divided into 6 groups containing 5 mice per group. On day 0, all mice were subcutaneously inoculated, into the region of the fat pad of the breast, with 2.5 × 10^5^ Tubo cells in 100 μl of PBS. On day 7, when tumors were palpable, mice were either left untreated (control), vaccinated in the flank with 1 × 10^6^ dendric cells in 100 μl of PBS, treated via i. p. injection with MK-2206 (50 mg/kg), IFN-γ (10 μg), MK-2206 plus IFN-γ, or MK-2206 plus dendritic cells. Dendritic cells were given 2 times per week and both MK-2206 and IFN-γ were given 5 times per week for 2 weeks with an intervening two-day rest between the two weekly cycles. Calipers were used to measure tumor size (length multiplied by width) every 2–3 days. Treatment was ceased on Day 18 after the mice had received a total of 10 treatments.

### Statistical analysis

To analyze the difference between treatment groups in the Alamar blue, trypan blue, TMRE, and Western blot assays treatment groups were normalized to a control of no treatment. A one-way ANOVA was used to determine if there was a significant difference between means and a Holm-Sidak test was used priori to compare the difference between individual groups. Sigma Plot software was used to run all statistical analyses. Treatment groups were considered significantly different if the *p* value was < 0.05.

## SUPPLEMENTARY MATERIALS


