# Supplementary material for: Th1 cytokines in conjunction with pharmacological Akt inhibition potentiate apoptosis of breast cancer cells in vitro and suppress tumor growth in vivo
Source: Oncotarget. 2020 Jul 28;11(30):2873–88. doi: 10.18632/oncotarget.27556 (PMC7392628; doi:10.18632/oncotarget.27556)
Supplement: Supplementary file 1 [file oncotarget-11-2873-s001.pdf]

Th1 cytokines in conjunction with pharmacological Akt inhibition potentiate apoptosis of breast cancer cells *in vitro* and suppress tumor growth *in vivo*

SUPPLEMENTARY MATERIALS

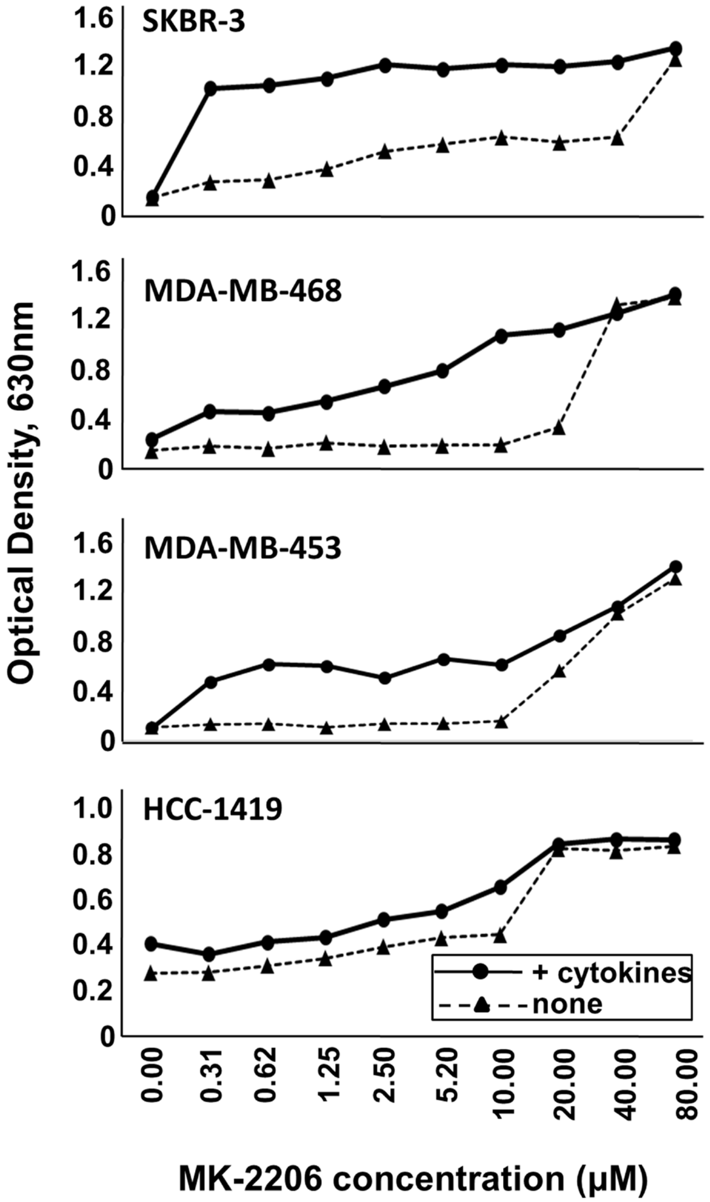

Supplementary Figure 1: MK-2206 dose response curves for four human breast cancer cell lines. SKBR-3, MDA-MB-468, MDA-MB-453 and HCC-1419 cells were cultured at 5× 10^3 per well in 96-well cluster plates overnight. The cells were then exposed to MK-2206 in serial dilutions ranging from 80 µM down to 0 µM either in the presence or absence of Th1 cytokines (IFN-γ and TNF-α at 10 ng/ml each), and incubated for an additional 72 h. Then, 20 µl of resazurin sodium salt solution (Alamar Blue dye; 1.4 mg/ml) was added per well, and cells incubated until color change occurred. Optical density of culture supernatants was determined at 630 nm.

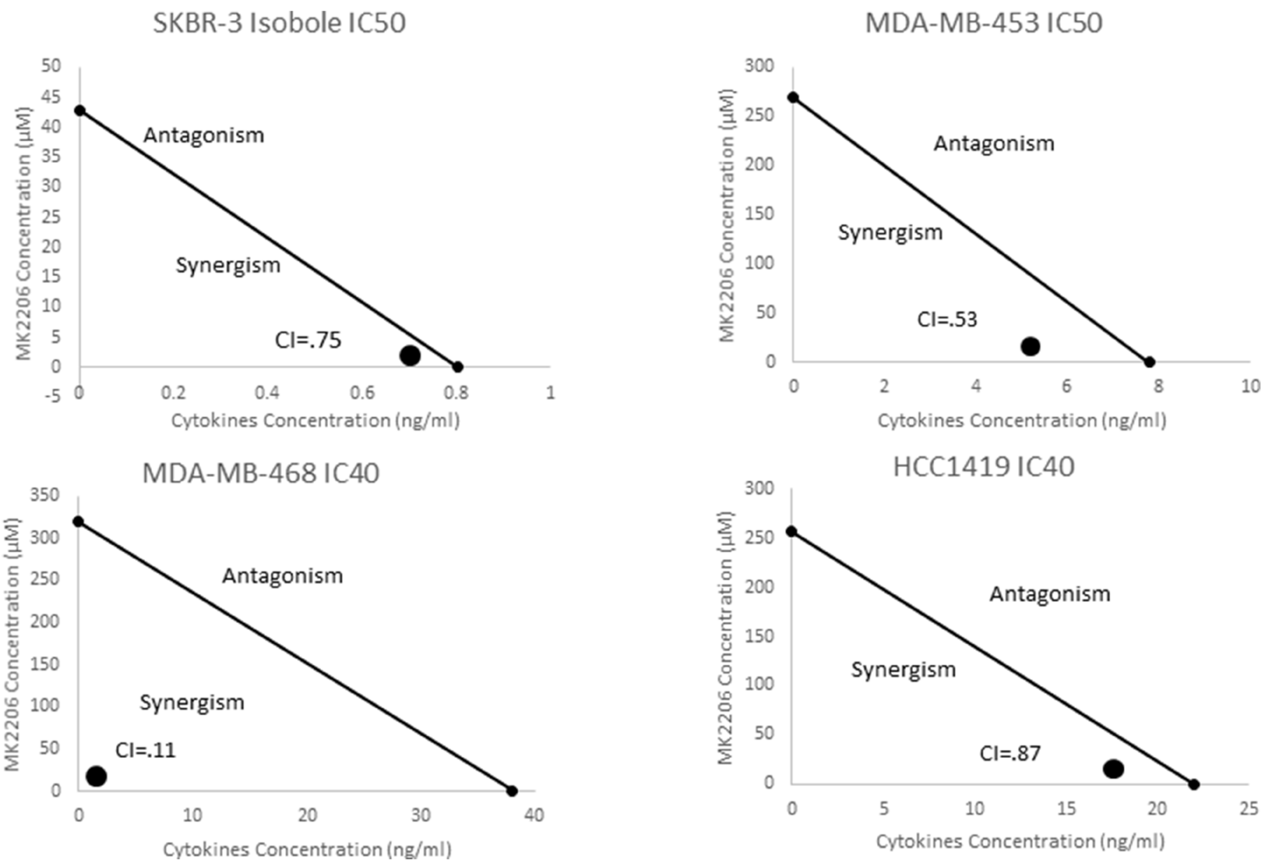

Supplementary Figure 2: Isoboles and corresponding CI value**s used to determine synergy between MK-2206 and Th1 cytokines in breast cancer cell lines.** SKBR-3, MDA-MB-468, MDA-MB-453 and HCC-1419 cells were cultured at 5×10^3 per well in 96-well cluster plates overnight. The cells were then exposed to either MK-2206 in serial dilutions ranging from 320 µM to 0 µM, Th1 cytokines (TNFα and IFNγ) in serial dilutions ranging from 320 ng/ml to 0 ng/ml of each cytokine, or both MK2206 and Th1 cytokines together in the same serial dilutions. The cells were incubated for an additional 72 h. Then, 20 µl of resazurin sodium salt solution (Alamar Blue dye; 1.4 mg/ml) was added per well, and cells incubated until color change occurred. Optical density of culture supernatants was determined at 630 nm. An IC_{50} (SKBR3 and MDA-MB-453) or an IC_{40} (MDA-MB-468 and HCC1419) value was generated by calculating the concentration of MK2206, cytokines, or both that gave 40 or 50% of the maximal effect of each treatment. The combination index (CI) was calculated using the IC values generated for each line.

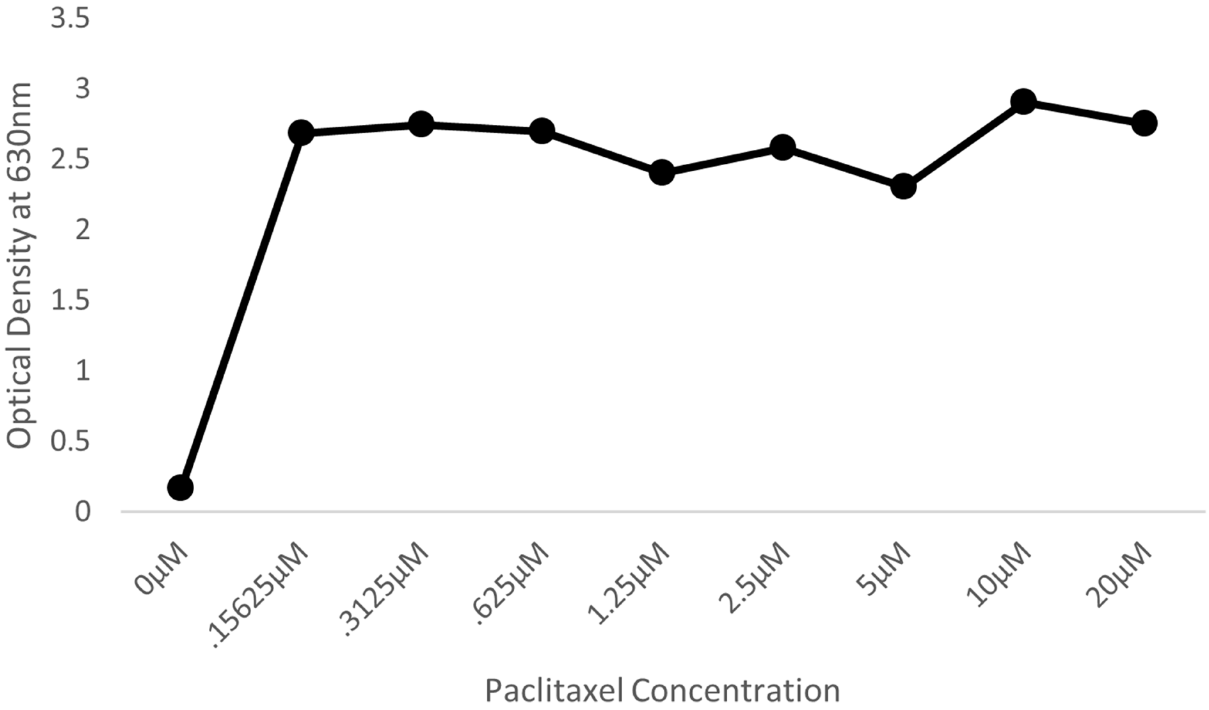

Supplementary Figure 3: Dose response curve for paclitaxel. SKBR-3 cells were cultured at 5×10^3 per well in 96-well cluster plates overnight. The cells were then exposed to paclitaxel in serial dilutions ranging from 20 μM to 0.15 μM and incubated for an additional 72 h. Then, 20 μl of resazurin sodium salt solution (1.4 mg/ml) was added per well, and cells incubated until color change occurred. Optical density of culture supernatants was determined at 630 nm.

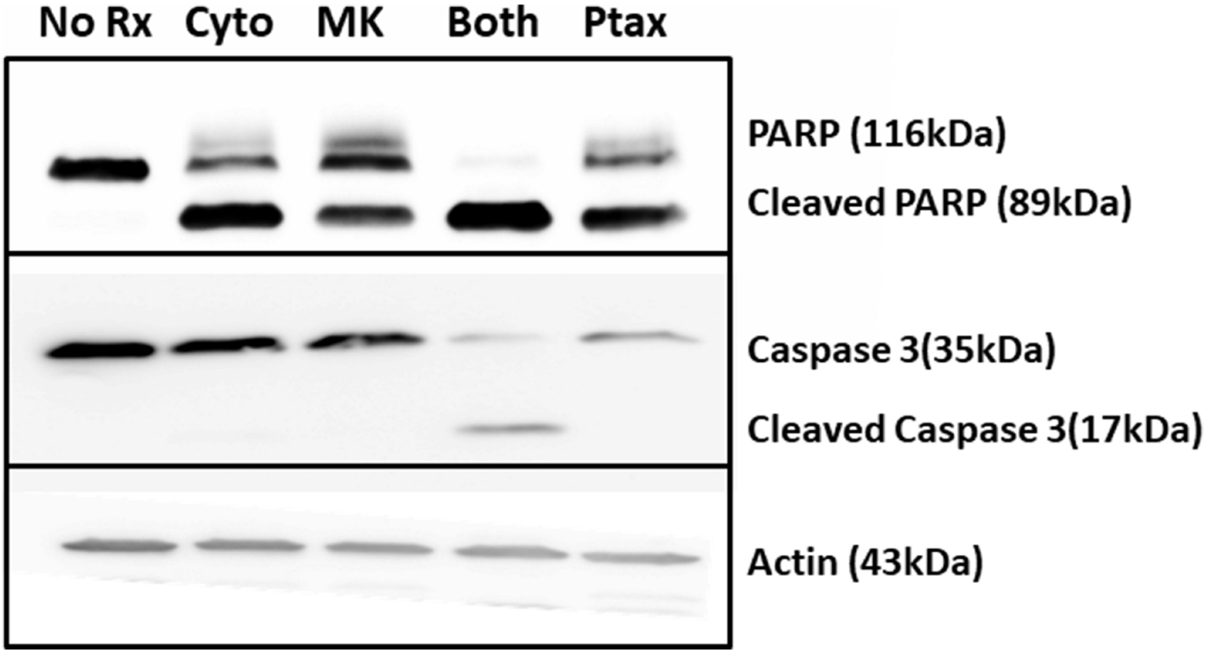

Supplementary Figure 4: Th1 cytokines in conjunction with MK-2206 potentiate cleavage of PARP and Caspase 3 in SKBR3 cells. SK-BR-3 cells were treated with either Th1 cytokines (TNF-α plus IFN-γ; 10 ng/ml), 10 µM MK-2206, Th1 cytokines plus MK-2206, paxlitaxel (300 nM) or left untreated (control). Between 24 and 48 hours of incubation the cells were harvested, extracted in presence protease and phosphatase inhibitors, and 30 µg/well total proteins were separated on a 4–15% gradient SDS-PAGE gel prior to electrotransfer onto PVDF membranes. Blots were then probed with HRP-conjugated anti-PARP or anti-caspase 3 antibodies, and bands detected using chemiluminescence. Blots were then probed with anti-actin to assess loading variability.

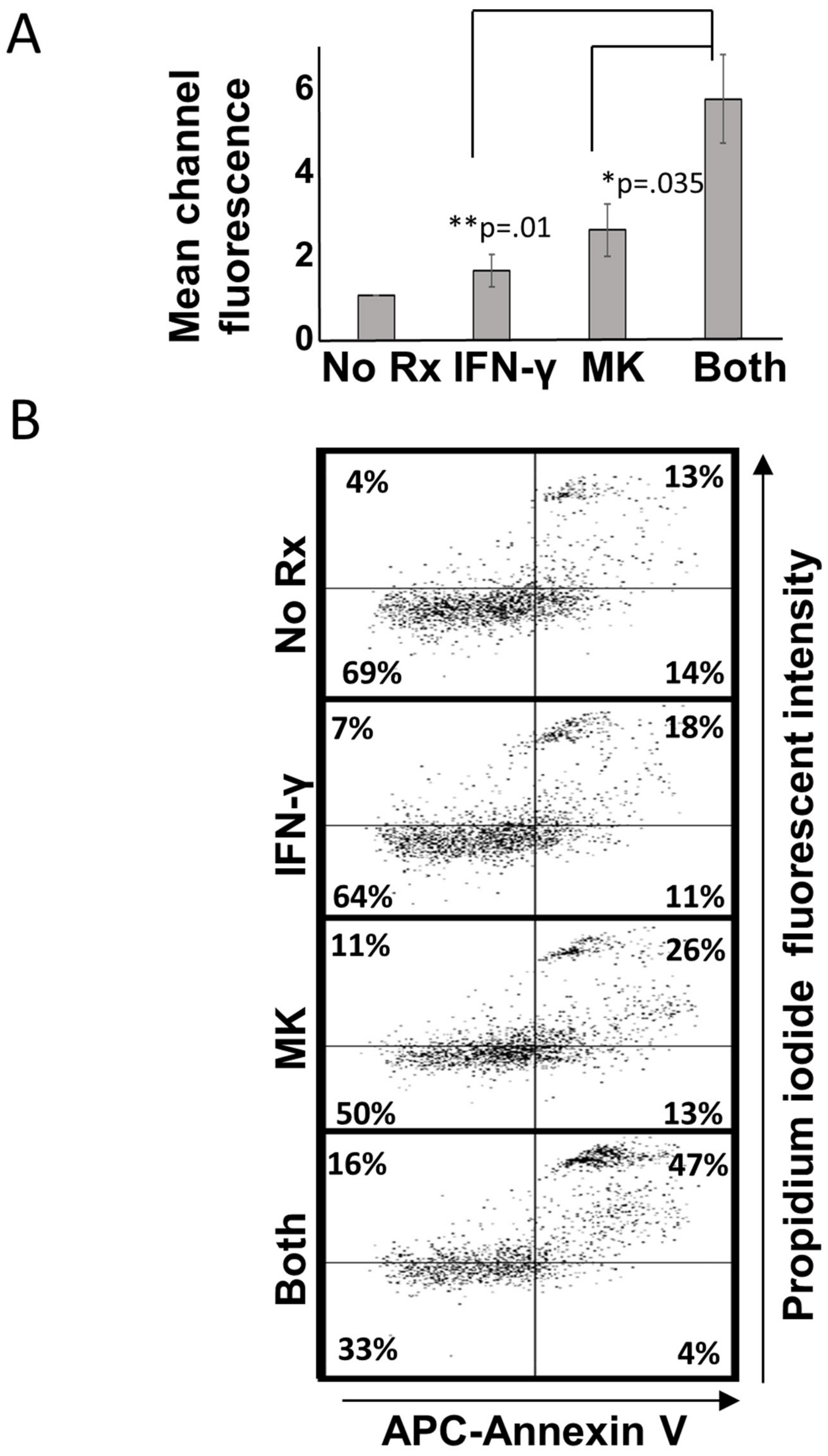

Supplementary Figure 5: The HCC-1419 human breast cancer cell line responds to MK-2206 plus interferon gamma in the absence of TNF alpha. HCC-1419 cells were cultured at 1×10^5 per well in 12-well cluster plates overnight. The following day cells were then exposed to IFN-γ (50 ng/ml each), MK-2206 (10 μM), both treatments, or left untreated. After 72 h further incubation, cells were then harvested, washed and (**A**) stained with Trypan Blue dye, then analyzed by flow cytometry for dye uptake. The bar graph represents composite data from at least 3 separate experiments. Error bars denote SEM. (**B**) stained with propidium iodide and APC-labeled Annexin V. Stained cells were subjected to flow cytometry and data evaluated by quadrant analysis, with double-staining cells (upper right quadrant) defined as apoptotic.
